# Dietary Yam (*Dioscorea opposita* Thunb.) Ameliorates Parkinson’s Disease in Mice via Gut Microbiota-Driven Mitochondrial Improvement and Neuroinflammation Inhibition

**DOI:** 10.3390/nu18081208

**Published:** 2026-04-11

**Authors:** Shuqing Zhang, Wenjia Pan, Chen Ma, Yinghua Luo, Li Dong, Junfu Ji, Lingjun Ma, Daotong Li, Fang Chen

**Affiliations:** College of Food Science and Nutritional Engineering, National Engineering Research Centre for Fruit and Vegetable Processing, Key Laboratory of Fruits and Vegetables Processing, Ministry of Agriculture, Engineering Research Centre for Fruits and Vegetables Processing, Ministry of Education, China Agricultural University, Beijing 100083, China; zhangshuqing@cau.edu.cn (S.Z.); pwj@cau.edu.cn (W.P.); machen21@cau.edu.cn (C.M.); luoyinghua@cau.edu.cn (Y.L.); li_dong127@163.com (L.D.); junfu.ji@cau.edu.cn (J.J.); lingjun.ma@cau.edu.cn (L.M.)

**Keywords:** Parkinson’s disease, Chinese yam (*Dioscorea opposita* Thunb.), gut microbiota, microbiota–gut–brain axis, purine metabolism, mitochondrial function

## Abstract

**Background/Objectives:** Parkinson’s disease (PD) is a progressive neurodegenerative disorder that poses a substantial threat to global human health. Yam (*Dioscorea opposita* Thunb.) is a traditional medicinal and edible plant that has long been used in Asia, Africa, and the Caribbean. Its major bioactive components, such as dioscin and polysaccharides, have been reported to exhibit neuroprotective effects; however, the impact of dietary yam on PD progression remains to be elucidated. Therefore, we sought to evaluate its neuroprotective potential and the underlying mechanisms in 1-methyl-4-phenyl-1,2,3,6 tetrahydropyridine (MPTP)-induced PD mice. **Methods:** Mice received six-week dietary yam supplementation. Behavioral, histological, and neurochemical analyses were performed to assess motor function, dopaminergic neuron integrity, and dopamine levels. Gut microbiota and metabolic profiles were analyzed using 16S rRNA gene sequencing and non-targeted metabolomics. Transcriptomic sequencing and Western blot analysis of the substantia nigra pars compacta (SNc) were conducted to investigate molecular mechanisms, and integrative multi-omics analysis was applied to explore microbiota–metabolite–host interactions. **Results:** Yam supplementation improved motor function, preserved nigrostriatal dopaminergic neurons, and restored striatal dopamine levels in PD mice. Notably, yam was associated with the maintenance of intestinal homeostasis by strengthening barrier integrity and enriching beneficial taxa, including *Ileibacterium*, *Lachnospiraceae NK4A136 group*, and *Blautia*. Consistently, yam also elevated neuroprotective purines and amino acids, including inosine, xanthine, and succinic acid. At the molecular level, yam treatment modulated mitochondrial oxidative phosphorylation by increasing PGC-1α and COX7c expression, and reduced inflammasome-related neuroinflammatory signaling. Integrative modeling showed significant associations between yam-modulated genes and PD-related indices with microbiota and metabolites. **Conclusions:** These findings suggest that yam may represent a potential dietary strategy for alleviating PD-related neurodegeneration by modulating the microbiota–gut–brain axis.

## 1. Introduction

Parkinson’s disease (PD) is the second common neurodegenerative disease posing a major health challenge to the elderly population. The prevalence of PD among individuals aged 65 is about 2–3%, with a tendency to rise in correlation with advancing age [1]. The clinical manifestations of PD are highly heterogeneous, mainly manifested as motor symptoms such as tremor, bradykinesia, rigidity and postural instability [2]. The main pathological features of PD are the death of dopaminergic neurons in the substantia nigra pars compacta (SNc) and the aggregation of α-synuclein (α-syn) [1]. These pathological changes lead to the occurrence of motor symptoms in PD patients. Although current dopamine replacement therapy can alleviate the motor symptoms of PD patients to a certain extent, they cannot prevent the continuous degeneration of dopaminergic neurons and are frequently associated with long-term side effects [3,4]. Therefore, it is particularly urgent and important to explore safe and effective treatments to cope with the increasing burden of PD within an aging demographic.

The association between neurological diseases and the central nervous system does not exist in isolation but is influenced by a combination of factors. In fact, emerging evidence reveals that intricate bidirectional signaling pathways between the gastrointestinal tract and the central nervous system [5]. Several studies have reported gut microbial dysbiosis in PD mouse models and PD patients, and found that the abundance and diversity of the gut microbiota in these subjects were significantly different compared with wild-type animals and control participants [6,7,8]. For instance, antibiotic treatment of transgenic mice overexpressing human α-syn, a typical transgenic mouse model of PD, reduced motor dysfunction and α-syn pathology [9]. Further research revealed that transferring gut microbiota from Thy1-aSyn mice to germ-free mice induced similar motor deficits and α-syn pathology, mirroring the neurodegenerative features of the donor mice [9]. This finding directly demonstrates the key role of the gut microbiota in the development of PD. The metabolites of gut microorganisms constitute a key entry point for dissecting their effects on brain physiological and pathological mechanisms. A groundbreaking PD study revealed that alterations in gut microbiota composition triggered a notable increase in gut microbiota-derived propionate levels in the colon, which activated FFAR3 receptors in the enteric nervous system and significantly alleviated PD symptoms [10]. In addition, other studies have also demonstrated that higher levels of short-chain fatty acids (SCFAs) inhibited activation of the TLR4/TNF-α signaling pathway, reduced overactivation of glial cells, and restored neurotransmitter levels, thereby exerting a neuroprotective effect on PD mice [8]. These findings suggest that the gut microbiota may be a key mediator in maintaining brain physiological function, and targeting the gut microbiome may be an effective treatment for the prevention and treatment of PD.

Dietary strategies, by profoundly modulating the taxonomic diversity and functional capacity of the gut microbiota, serve as a critical regulator of host health. Chinese yam (*Dioscorea opposita* Thunb.) is a perennial herb widely distributed in Asia and Africa. Its tuberous roots are rich in a variety of bioactive compounds, including proteins, polysaccharides, dietary fiber, dioscin, and allantoin, which collectively confer significant health benefits [11]. Recent advances in phytochemistry and pharmacology have unveiled the multifaceted biological activities of yam, particularly its antioxidant, anti-inflammatory, and immunomodulatory properties [11]. Notably, emerging evidence suggests that yam-derived bioactive compounds can profoundly influence gut microbiota homeostasis. For example, Chinese yam polysaccharides were metabolized by gut microbiota, promoting SCFAs production and the enrichment of beneficial taxa including *Bifidobacterium* and *Megasphaera* in an in vitro fermentation model [12]. In colorectal cancer models, yam polysaccharides intervention significantly reconfigures the gut microbial landscape by enriching beneficial taxa such as *Clostridia_UCG-014* and *Actinobacteria* while suppressing pathogenic groups like *Enterorhabdus* and *Desulfovibrionaceae* [13]. This microbial remodeling further rectifies host metabolic dysregulation, specifically within the tryptophan and purine pathways, thereby mitigating systemic inflammation. Moreover, yam proteins have also been shown to improve the imbalance of intestinal microbial homeostasis by increasing the relative of beneficial bacteria such as *Lactobacillus* in the microbiome and reducing the levels of *Desulfovibrio* and *Helicobacter* [14]. Beyond its impact on gut health, accumulating evidence indicates that yam-derived bioactive constituents, including diosgenin and dioscin, hold promise for mitigating neurodegenerative diseases, including Alzheimer’s disease, cognitive impairment, and depression [15,16,17]. While individual yam-derived compounds have been investigated, the neuroprotective potential of whole dietary yam, particularly in relation to the microbiota–gut–brain axis, remains largely unexplored. Therefore, we hypothesized that dietary yam may alleviate PD-related pathology through modulation of the gut microbiota and associated metabolic pathways.

This study systematically evaluated yam in PD model from a holistic dietary intervention perspective. Through behavioral assessments and dopaminergic neuronal integrity analysis, the effects of dietary yam on PD-related motor disorders and neuropathological changes were comprehensively evaluated. To further elucidate the potential mechanisms of gut–brain interaction, this study constructed an integrated multi-omics research framework, jointly conducting gut microbiota 16S rRNA sequencing, colonic contents metabolomics analysis and SNc transcriptomic analysis, to explore the underlying mechanisms by which dietary yam regulates the gut microbiota–metabolite–host molecular signaling axis and thus influences the pathological process of PD. Overall, this study suggests that yam may represent a potential dietary intervention strategy for alleviating PD-related neurodegeneration by modulating the microbiota–gut–brain axis.

## 2. Material and Methods

### 2.1. Animals and Experimental Design

A total of 30 eight-week-old male C57BL/6J mice were obtained from Beijing Vital River Laboratory Animal Technology Co., Ltd. (Beijing, China) and were housed in the specific pathogen-free (SPF) facility of the Laboratory Animal Center, China Agricultural University. Mice were maintained under controlled environmental conditions (12 h light/dark cycle, 22–24 °C, 50–60% relative humidity) with ad libitum access to food and water. All procedures involving animals followed the Regulations for the Administration of Affairs Concerning Experimental Animals of Beijing Municipality and were approved by the Institutional Animal Care and Use Committee of China Agricultural University (IACUC Approval No.: AW41112202-4-1).

The preparation of yam powder for dietary intervention was performed using a standardized protocol based on previous study [18,19]. Yam tubers (*Dioscorea opposita* Thunb., “Tiegun yam”) were purchased from Xincheng Huaiyao Co., Ltd. (Jiaozuo, China), a well-recognized geographical origin of this cultivar. Fresh tubers were thoroughly washed and steamed for 40 min to ensure complete starch gelatinization. The samples were then cooled, sliced, freeze-dried, and ground into a fine powder. The powder was further passed through a 100-mesh sieve to ensure uniform particle size. All processing steps were conducted under controlled and standardized conditions to minimize batch variability and ensure consistency with previously characterized yam materials. The final yam powder was sealed and stored at −20 °C until use.

To evaluate the neuroprotective effect of dietary yam supplementation on a PD mouse model, 8-week-old male C57BL/6J mice were acclimatized for one week and then randomly assigned to three groups (*n* = 10 per group) using a randomized block design based on baseline body weight to ensure comparable physiological conditions across groups: control group (normal diet), MPTP group (normal diet), and MPTP + Yam group (10% (*w*/*w*) yam powder added to the basal diet), fed continuously for 6 weeks. The dosage of yam was determined based on previous literature reports and preliminary experimental results [18,20]. The sample size (*n* = 10 per group) was determined based on commonly used experimental designs for the MPTP-induced PD model [21,22,23,24], and was considered sufficient to support multiple primary readouts, including behavioral, histological, neurochemical, and multi-omics analyses. Male mice were used in this study to reduce biological variability and maintain experimental consistency, particularly in behavioral and neurochemical assessments in the MPTP-induced PD model. PD model induction began in second week of dietary intervention. Mice in the MPTP group and MPTP + Yam group were intraperitoneally injected with MPTP (30 mg/kg body weight/day) for five consecutive days, while mice in the control group were injected with an equal volume of physiological saline.

At the end of the intervention, motor performance was evaluated using a battery of behavioral tests, including the pole test, rotarod test, inverted grid test, and hindlimb clasping test. These behavioral assessments were conducted in vivo in live animals prior to sacrifice. Following behavioral assessment, mice were anesthetized, and blood samples were collected via retro-orbital bleeding. Serum was isolated by centrifugation and stored at −80 °C for subsequent ELISA-based quantification of inflammatory cytokines and circulating lipopolysaccharide (LPS). Thereafter, mice were euthanized by cervical dislocation, and tissues, including the SNc, striatum, colon, and cecal/fecal contents, were harvested, snap-frozen in liquid nitrogen, and stored at −80 °C for downstream multi-omics profiling (transcriptomics, 16S rRNA sequencing, untargeted metabolomics) and molecular assays. All subsequent histological, molecular, biochemical, and multi-omics analyses were performed on serum or tissues collected after euthanasia.

### 2.2. Behavioral Tests

To comprehensively assess the effects of yam on motor function of MPTP-treated mice, the pole test, the rotarod test, the inverted grid test, and the hindlimb test were conducted as described previously [9,25]. All behavioral assessments were performed by experimenters blinded to group allocation to minimize potential bias. All experiments were conducted under standardized laboratory conditions. Prior to testing, each mouse was allowed to acclimate to the experimental room for one hour to minimize the impact of environmental changes. In addition, to prevent residual odors from influencing behavior, all apparatus were thoroughly cleaned and disinfected with 75% ethanol before each test.

#### 2.2.1. Pole Test

The Pole Test was used to evaluate balance and motor coordination. Specifically, the apparatus consists of a 55 cm high, 1 cm diameter vertical pole with adhesive tape to provide a rough surface to prevent the mouse from slipping, the bottom fixed to a base for stability, and a spherical platform fixed on the top. Each mouse was placed on the platform at the top of the pole, with its head oriented downward, and allowed to descend. The total time required for the mouse to fully descend from the top of the pole and for one of its hind legs to fully reach the bottom of the pole was recorded. Three consecutive trials were performed per mouse, with an interval of approximately 10 min between trials, and the mean descent time was used as the outcome measure.

#### 2.2.2. Rotarod Test

The rotarod test primarily assessed balance, coordination, and endurance, and was divided into a training phase and a formal testing phase. During the training period, mice underwent two trials per day for three consecutive days using a rotarod operating at a constant speed of 5 rpm to familiarize them with the apparatus. In the formal testing period, each mouse was placed on the rotarod, which accelerated gradually from 5 rpm to 30 rpm over a 5 min period. The latency to fall was recorded for each trial. Each mouse performed three trials, with an interval of approximately 15 min between tests, and the average fall latency was calculated as the final measure.

#### 2.2.3. Inverted Grid Test

The inverted grid test was designed to assess grip strength and muscle endurance. The mouse was placed in the center of a 30 cm × 30 cm horizontal metal screen composed of 1 mm diameter wire with a 1 cm mesh size. The grid was placed on a stand 40 cm above an open cage with soft bedding to prevent injury. The grid was then quickly inverted to force the mouse into a hanging posture. The time the mouse maintained the grip was recorded (maximum observation time was 180 s). Three trials were performed for each mouse, with an interval of approximately 10 min between each trial, and the average hanging time was used as the final result.

#### 2.2.4. Hindlimb Scoring

During the hindlimb scoring test, mice were suspended by their tails (gripped near the base) and their hindlimb posture was observed for 10 s. Then the score was assigned according to the degree of hindlimb retraction, as follows:

0: Both hindlimbs were fully extended outward and showed normal free movement;

1: Only one hindlimb was extended outward and moved freely;

2: Both hindlimbs were retracted inwards, but some degree of flexibility is retained;

3: Both hindlimbs were fully retracted with no observable movement;

Each mouse was tested independently three times, with approximately 10 min between each test. The final hindlimb score for each animal was determined by calculating the average of the three test results.

### 2.3. Enzyme-Linked Immunosorbent Assay

Serum tumor necrosis factor-α (TNF-α), interleukin (IL)-1β, IL-6, IL-18, IL-10, and LPS levels were determined using an enzyme-linked immunosorbent assay (ELISA) kit (Meimian Industrial Limited by Share Ltd., Yancheng, China). The experimental procedures were carried out according to the manufacturer’s instructions. The TNF-α, IL-1β, IL-6, IL-18, IL-10, and LPS levels were determined by measuring the absorbance using a microplate reader and plotting a standard curve.

### 2.4. Hematoxylin and Eosin (H&E) Staining and Alcian Blue–Periodic Acid Schiff (AB-PAS) Staining

The H&E and AB-PAS staining for the colon section were performed as our previously study [26]. Colonic tissue was collected and fixed in 4% paraformaldehyde and embedded in paraffin. The embedded colonic tissue was cut into 4 μm sections and stained using a hematoxylin–eosin (H&E) HD constant dye kit and AB-PAS staining kit (Wuhan Servicebio Biotechnology Co., Ltd., Wuhan, China) according to the manufacturer’s instructions. Images were acquired using a Pannoramic MIDI tissue section digital scanner and Caseviewer 2.4 software. The crypt length, muscle layer thickness, goblet cell number, and mucus lining thickness were measured using Image-J software (Version 1.25a, National Institutes of Health, Bethesda, MD, USA).

### 2.5. Immunofluorescence and Immunohistochemical Staining

Mouse brain and colon tissues were collected for immunohistochemistry and immunofluorescence staining according to previous study [25]. For immunofluorescence staining, paraffin sections of brain and colon tissues were blocked with 10% BSA for 30 min at room temperature after antigen retrieval to reduce nonspecific binding. Brain tissue sections were incubated with TH primary antibody (Proteintech Group, Inc., Wuhan, China), and colon tissue sections were incubated with ZO-1 and Claudin-3 primary antibodies (Proteintech Group, Inc., Wuhan, China) at 4 °C overnight. The next day, after washing with PBS (pH 7.4), the sections were incubated with HRP-conjugated secondary antibodies of the corresponding species of the primary antibody for 2 h in the dark. Then, the corresponding TSA was added to the sections for 10 min in the dark and the nuclei were counterstained with DAPI. Finally, the sections were treated with an autofluorescence quencher and mounted.

For immunohistochemical staining, paraffin sections of brain tissue were incubated with anti-TH primary antibody at 4 °C overnight. Sections were washed three times with PBS (pH 7.4) and covered with HRP-conjugated secondary antibody for 1 h. Then, brain tissue sections were stained with 3,3′-diaminobenzidine (DAB) and counterstained with hematoxylin for nuclei. Finally, sections were dehydrated and mounted. Images were acquired using a Pannoramic MIDI tissue section digital scanner (3DHISTECH Ltd., Budapest, Hungary) and Caseviewer software (Version 2.4.0. 119028, 3DHISTECH Ltd., Budapest, Hungary). Further quantitative analysis was performed using Image-J software (Version 1.25a, National Institutes of Health, Bethesda, MD, USA).

### 2.6. Western Blot

Western blotting analysis of brain SNc tissue was performed as previously described [27]. Briefly, RIPA lysis buffer containing protease and phosphatase inhibitors (100:1) was added to the well-ground SNc tissue. The homogenates were lysed on ice for 30 min, centrifuged at 13,000× *g* for 10 min at 4 °C, and the supernatant was collected. Protein concentration was quantified using the BCA assay, and all samples were adjusted to equal concentrations. Proteins were mixed with 5× SDS sample buffer at a 4:1 ratio, denatured at 100 °C for 10 min, and stored at −80 °C.

Protein samples were separated using SDS-PAGE and then transferred onto PVDF membranes activated with methanol. PVDF membranes were blocked with TBST-diluted 5% skim milk powder for 90 min, followed by overnight incubation at 4 °C with primary antibodies, including TH (25859-1-AP, Proteintech, Wuhan, China, 1:10,000), DAT (22524-1-AP, Proteintech, Wuhan, China, 1:1000), COX7c (11411-2-AP, Proteintech, Wuhan, China, 1:1000), PGC1α (NBP1-04676, Novus Biologicals, Centennial, CO, USA, 1:500), p-CREB (9198, CST, Danvers, MA, USA, 1:1000), CREB (9197, CST, Danvers, MA, USA, 1:1000), NLRP3 (ab263899, Abcam, Cambridge, UK, 1:1000), Caspase-1 (83383, CST, Danvers, MA, USA, 1:1000), Cleavage Caspase-1 (PA5-99390, Thermo Fisher, Waltham, MA, USA, 1:500), and β-actin (ab8226, Abcam, Cambridge, UK, 1:2000). The next day, the membranes were washed three times with TBST buffer and incubated with HRP-conjugated secondary anti-rabbit (32,460, Thermo Fisher, Waltham, MA, USA, 1:3000) or anti-mouse (31,430, Thermo Fisher, Waltham, MA, USA, 1:10,000) antibodies at room temperature for 1 h. Immunoreactive bands were visualized using ECL chemiluminescence reagents and captured with a ChemiDoc Touch Imaging System (Bio-Rad, Hercules, CA, USA). Protein grayscale analysis was performed using Image-J software (National Institutes of Health, Bethesda, MD, USA).

### 2.7. 16S rRNA Sequencing Analysis

Fecal pellets were collected from mice and immediately placed in sterile EP tubes. All fecal samples were immediately frozen and stored at −80 °C for further analysis. To evaluate the effects of yam treatment on the gut microbiota of MPTP-induced PD mice, 16S rRNA gene sequencing was performed on fecal samples as described previously [28,29]. Briefly, microbial genomic DNA was extracted from fresh fecal samples using the QIAamp Fast DNA Stool Mini Kit (Qiagen, Hilden, Germany) following the manufacturer’s instructions, followed by quality and quantity assessment through agarose gel electrophoresis and NanoDrop spectrophotometry. The V3–V4 hypervariable regions of the 16S rRNA gene were amplified using specific primers 341F (5′-CCTACGGGNGGCWGCAG-3′) and 802R (5′-TACNYGGGTATCTAATCC-3′). PCR conditions were as follows: initial denaturation at 95 °C for 3 min, followed by 30 cycles of denaturation at 98 °C for 20 s, annealing at 58 °C for 15 s, and extension at 72 °C for 20 s, with a final extension at 72 °C for 5 min. Purified amplicons were subjected to paired-end 300 bp sequencing on the Illumina MiSeq platform (Illumina, San Diego, CA, USA).

Raw sequencing data were quality-filtered using PANDAseq (v2.9), removing sequences with an average Phred quality score below 20, those containing more than 3 ambiguous bases, and retaining reads between 250 and 500 bp. Operational taxonomic units (OTUs) were clustered at 97% similarity using USEARCH (v7.0.1090) and annotated against the Ribosomal Database Project (RDP) or Greengenes database. All samples were processed using the same DNA extraction and sequencing workflow to reduce technical variability. As all samples were analyzed within a single workflow, no explicit batch-effect correction was applied. Alpha diversity indices (Shannon and Simpson) were calculated using QIIME (v1.9.1), while beta diversity was assessed through PCoA based on weighted UniFrac distances. The consistency of microbial profiles across samples was tested using Adonis and ANOSIM analyses. Differential microbial taxa were identified using ANCOM (FDR-adjusted *p* < 0.05), and key biomarkers were determined through LEfSe (LDA score > 3). Spearman correlation analysis was used to detect the correlation between differentially expressed bacterial genera and Parkinson’s characteristic pathological indicators. All statistical analyses were performed in R (v3.5.1), and data visualization was conducted using ggplot2 (4.0.2, R Foundation for Statistical Computing, Vienna, Austria).

### 2.8. Untargeted Metabolomics Sequencing Analysis

To investigate the changes in gut microbial metabolites, fecal samples from experimental animals were subjected to untargeted metabolomics analysis [29]. After collecting fecal samples from each group of mice, they were rapidly placed in −80 °C storage. Samples were processed by ultrasonication in methanol, followed by centrifugation to collect the supernatant, which was then filtered through a 0.22 μm filter membrane for subsequent testing. Chromatographic separation was achieved using an ACQUITY UPLC HSS T3 column (100 mm × 2.1 mm, 1.8 μm) (Waters Corporation, Milford, MA, USA) with a column temperature of 50 °C and a flow rate of 0.4 mL/min. Mobile phase A consisted of 0.1% formic acid in water, and mobile phase B consisted of 0.1% formic acid in acetonitrile. The gradient elution program was as follows: 0–2 min, 100% A; 2–11 min, 0–100% B; 11–13 min, 100% B; 13–15 min, 0–100% A. Mass spectrometry detection was performed using a Xevo G2 XS QTOF (Waters Corporation, Milford, MA, USA) mass spectrometer in both positive and negative ion modes. In positive ion mode, the capillary voltage was 3.0 kV and the cone voltage was 40 V; in negative ion mode, the capillary voltage was 2.0 kV and the cone voltage was 40 V. The mass range was set from 50 to 1200 *m*/*z*, and the scan time was 0.2 s.

In terms of data processing, Progenesis QI software (Version 2.3, Nonlinear Dynamics, Newcastle upon Tyne, UK) was utilized for feature extraction, alignment, and normalization. Metabolites were identified by combining online databases and literature. Multivariate statistical analysis methods, including Partial Least Squares Discrimination Analysis (PLS-DA) and orthogonal partial least squares discriminant analysis (OPLS-DA), were employed to identify differential metabolites. The criteria for differential metabolites were VIP > 1 and FDR-adjusted *p*-values < 0.05. All statistical analyses were completed on the R (v3.5.1) platform, and data visualization was accomplished using ggplot2 package.

### 2.9. Transcriptome Sequencing Analysis

To uncover the molecular basis of the neuroprotective effects of yam treatment on MPTP-induced PD mice, RNA sequencing was performed on the SNc of mice as previously described with modifications [30,31]. SNc tissues were dissected and immediately snap-frozen in liquid nitrogen. Total RNA was extracted from the SNc tissue, and the concentration and purity of the extracted RNA were tested by Nanodrop2000 spectrophotometer (Thermo Fisher Scientific, Waltham, MA, USA), the integrity of RNA was tested by agarose gel electrophoresis, and the RNA quality number (RQN) value was determined by Agilent5300 (Agilent Technologies, Santa Clara, CA, USA). The total RNA amount required for a single library construction is 1 μg, the concentration is ≥30 ng/μL, RQN > 6.5, and OD260/280 is between 1.8 and 2.2. Subsequently, high-quality RNA samples underwent mRNA enrichment using oligo (dT) magnetic beads.

The enriched mRNA was then fragmented by heating at 94 °C for 5 min, and first-strand cDNA synthesis was performed with random hexamer primers. This was followed by second-strand synthesis to generate double-stranded cDNA, which was purified using AMPure XP beads (Beckman, Brea, CA, USA). The purified cDNA was end-repaired, A-tailed, and ligated with Illumina sequencing adapters. After PCR amplification and quality control via Qubit (Thermo Fisher Scientific, Waltham, MA, USA) and Bioanalyzer (Agilent, Carpinteria, CA, USA), the final libraries were sequenced on an Illumina NovaSeq6000 platform using paired-end sequencing. Post-sequencing, adapter sequences and low-quality bases were trimmed using Trimmomatic, and the resulting clean reads were aligned to the *Mus musculus* reference genome (GRCm38) using HISAT2. Gene expression levels were quantified with featureCounts. Differential expression analysis was carried out using DESeq2. Genes with |log2 Fold Change| > 1 and PDR < 0.05 were considered significantly differentially expressed. To ensure reproducibility and minimize technical variability, all samples were processed using a unified experimental workflow under consistent conditions. No additional batch-effect correction was applied, as no separate technical batches were introduced.

Principal component analysis (PCA) was used to evaluate the differences between groups and the biological repetition of samples within the group. Volcano plot was used to visualize the distribution of DEGs. Gene Ontology (GO) and Kyoto Encyclopedia of Genes and Genomes (KEGG) enrichment analysis was performed on DEGs in each comparison using the hypergeometric test, with significance defined as FDR < 0.05. All statistical analyses were performed in R (v3.5.1) platform, and data visualization was performed using ggplot2package.

### 2.10. Integrated Multi-Omics Analysis

An integrated multi-omics analysis was performed to elucidate the regulatory mechanisms of yam using R software [31,32]. For individual omics datasets, rdCV-PLSDA was employed to construct predictive models. This framework, involving 200 repetitions and permutation analysis (*n* = 1000), was used to minimize overfitting and ensure the robustness of the identified signatures.

To integrate data across different platforms (e.g., transcriptomics and microbiomics), the DIABLO (Data Integration Analysis for Biomarker discovery using a Latent component method for Omics) framework was implemented. We utilized a full design matrix to identify variables from each dataset that exhibited maximal correlation. A tuning procedure was applied to select the optimal number of key variables based on the minimum misclassification rate, and the final model performance was validated using ten-fold cross-validation.

### 2.11. Statistical Analysis

With the exception of SNc transcriptome sequencing, gut microbiome sequencing, and fecal metabolome sequencing data, the remaining data were expressed as mean ± SEM. Data statistical analysis was performed using GraphPad Prism (version 9) software. The significance of data was assessed using the unpaired two-tailed Student’s t-test or one-way ANOVA followed by the post hoc Tukey’s multiple comparison tests. Statistical significance is indicated as follows: ns, not significant; *, *p* < 0.05; **, *p* < 0.01; and ***, *p* < 0.001. Omics-specific bioinformatic and statistical analyses are described in their respective subsections because the analytical pipelines, software tools, and significance criteria differ substantially across transcriptomic, microbiome, metabolomic, and integrative multi-omics datasets.

## 3. Results

### 3.1. Yam Protects Against Motor Deficits and Loss of Dopaminergic Neurons in MPTP-Induced PD Mice

**Figure 1:**
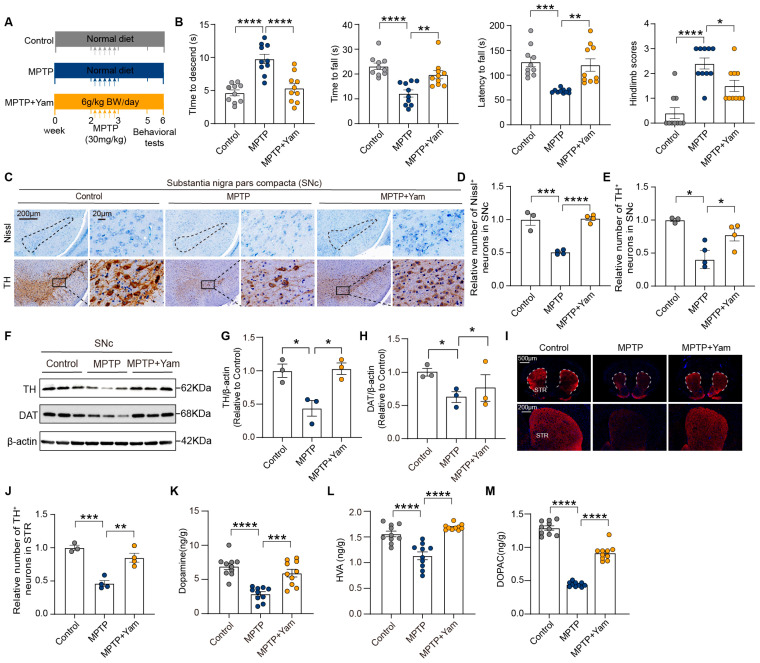
Dietary yam supplementation ameliorates motor deficits and dopaminergic neuron loss of MPTP-induced Parkinson’s disease mice. (**A**) Diagram of the experimental design; the arrows indicate the five intraperitoneal MPTP injections. (**B**) Time to descend the pole, retention duration on an inverted grid, latency to fall from an accelerating rotarod, and hindlimb scores evaluated based on the degree of inward contraction (*n* = 10 per group). (**C**) Representative photomicrographs of TH immunohistochemistry and Nissl staining in the SNc; the dashed line indicates the location of the SNc. (**D**,**E**) Quantitative analysis of TH^+^ neurons and Nissl^+^ cells in the SNc (*n* = 3–4 slices per group). (**F**) Representative Western blot bands of TH, dopamine transporter (DAT), and β-actin in the SNc. (**G**,**H**) Quantification of TH and DAT protein levels in the SNc (*n* = 3 per group). (**I**) Representative immunofluorescence images of TH (red) in the striatum; nuclei are counterstained with DAPI (blue). The dashed line indicates the location of the striatum. (**J**) Quantitative analysis of TH protein levels in the striatum (*n* = 3–4 slices per group). (**K**–**M**) Striatal concentrations of dopamine, homovanillic acid (HVA), and 3,4-dihydroxyphenylacetic acid (DOPAC) (*n* = 10 per group). Different colored dots represent individual data points. Data are performed as the mean ± SEM. * *p* < 0.05, ** *p* < 0.01, *** *p* < 0.001, and **** *p* < 0.0001. *p*-values were calculated by one-way ANOVA with Tukey’s or Tamhane’s tests.

The potential neuroprotective effect of yam on MPTP-induced chronic Parkinson’s symptoms was evaluated by daily oral administration of yam for six weeks (Figure 1A). Behavioral phenotyping showed that yam shortened the time to descend pole, prolonged retention duration on accelerating rotarod and grid apparatus, and improved the hindlimb clasping reflex score in PD mice, indicating that yam effectively alleviated the MPTP-induced impairment of motor coordination and balance (Figure 1B). The progressive degeneration of dopaminergic neurons within the SNc represents a cardinal neuropathological hallmark of PD [33]. Histopathological quantification showed the number of Nissl-positive cells and tyrosine hydroxylase (TH)-positive cells in the SNc of MPTP mice was significantly reduced by 48.4% and 69.7%, respectively, compared with control mice, which were both improved by yam supplementation (Figure 1C–E). Consistent with the results of pathological section analysis, Western blot analysis also revealed that yam can increase the levels of TH and dopamine transporter in the SNc of MPTP mice (Figure 1F–H). Moreover, Yam increased the expression of TH in the striatum of MPTP mice (by 45%) and effectively improved the depletion of dopamine and its metabolites 3,4-dihydroxyphenylacetic acid (DOPAC) and Homovanillic acid (HVA) (Figure 1I–M). These results indicate that dietary supplementation with yam alleviates MPTP-induced motor behavior disorders and dopaminergic neuron damage.

### 3.2. Yam Ameliorates Intestinal Barrier Dysfunction in MPTP-Induced PD Mice

**Figure 2:**
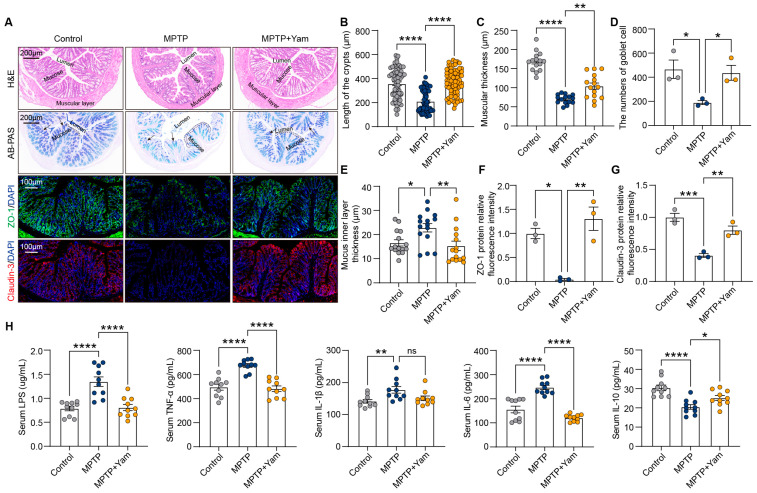
Dietary yam supplementation inhibits intestinal barrier dysfunction of MPTP-induced PD mice. (**A**) Representative images H&E staining, AB-PAS, and the immunochemical staining of ZO-1 (green) and claudin-3 (red) of the colon; nuclei are counterstained with DAPI (blue). In AB-PAS staining, dashed lines indicate the mucus inner layer region, and arrows indicate mucus-secreting goblet cells. (**B**,**C**) Quantification of crypt length and muscular thickness of colon tissue based on H&E staining (*n* = 3 mice per group). For crypt length analysis, all crypts in each slice of each group of mice were measured one by one, at least 63 crypts per slice. For analysis of muscular layer thickness, each colon tissue section was divided into 5 equal parts to measure the muscular layer thickness of each part to reduce the error. (**D**,**E**) Quantification of goblet cell number and mucus layer thickness based on AB-PAS staining (*n* = 3 mice per group). The blue mass pointed by the black arrow in the AB-PAS staining section is the stained goblet cell. The middle part of the double dotted line represents the inner mucus layer, and each colon tissue section was also divided into 5 equal parts to measure the inner mucus layer thickness of each part. (**F**,**G**) Fluorescence quantitative analysis of ZO-1 and claudin-3 proteins (*n* = 3 mice per group). (**H**) Level of LPS, TNF-α, IL-1β, IL-6, and IL-10 in serum (*n* = 10 mice per group). Different colored dots represent individual data points. Data of (**B**–**H**) presented as mean ± SEM. * *p* < 0.05, ** *p* < 0.01, *** *p* < 0.001, and **** *p* < 0.0001. ns, not significant. *p*-values were calculated by one-way ANOVA with Tukey’s or Tamhane’s tests.

Extensive research confirms that the widespread impairment of intestinal integrity in PD patients facilitates the systemic influx of pro-inflammatory microbial products, thereby driving the neuroinflammatory cascade via the gut–brain axis [34,35]. Therefore, we evaluated the effect of dietary yam supplementation on the integrity of the intestinal barrier by performing H&E staining of colon tissues. The crypt length and muscle layer thickness of MPTP-treated mice were significantly decreased compared with the control group mice, while yam supplementation reversed the structural damage of intestinal tissue (Figure 2A–C). When we used AB-PAS to stain the distal intestinal tissue, we found that yam increased the number of goblet cells and the thickness of the mucus inner layer in MPTP mice (Figure 2A,D,E), suggesting that it may be related to the improvement of the intestinal inflammatory environment [36]. We also found that yam supplementation increased the expression levels of tight junction proteins ZO-1 and Claudin-3 in MPTP mice (Figure 2A,F,G). To further confirm the effect of yam on the integrity of the intestinal barrier, we measured the levels of LPS and inflammatory cytokines in the serum. ELISA analysis showed that yam intervention significantly reduced the levels of LPS and pro-inflammatory cytokines TNF-α and IL-6 in serum of MPTP mice (Figure 2H). The serum level of pro-inflammatory cytokine IL-1β showed a downward trend upon yam intervention, although the difference was not significant (Figure 2H). In addition, yam intervention inhibited the MPTP-induced decrease in anti-inflammatory cytokine IL-10 (Figure 2H). Taken together, these findings suggest that dietary supplementation with yam improves MPTP-induced intestinal barrier dysfunction to dampen systemic inflammation.

### 3.3. Yam Reshapes the Diversity and Composition of the Gut Microbiota in MPTP-Induced PD Mice

**Figure 3:**
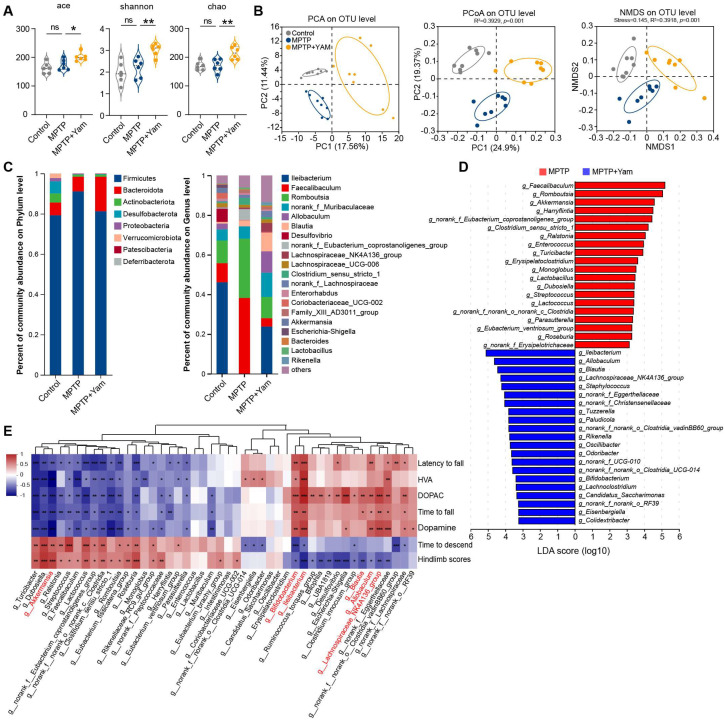
Dietary yam supplementation alleviates fecal microbiota dysbiosis of MPTP-induced PD mice. (**A**) Analysis of alpha diversity of gut microbiota by Ace, Shannon, and Chao index analysis (*n* = 8 mice per group). (**B**) PCA based on the relative abundance of OTUs at the genus level using the Bray–Curtis dissimilarity matrix, PCoA based on weighted UniFrac distance and Permutational MANOVA (adonis) was used to examine the difference in sample gut microbiota community composition (the colored ellipse denotes an 80% confidence within each group), and NMDS based on the binary_jaccard distance plot was employed to test the difference between different samples (the colored ellipse denotes an 80% confidence within each group) (*n* = 8 mice per group). (**C**) Relative abundance of gut microbiota at the phylum and the genus levels among Control, MPTP, and MPTP + Yam group mice. (**D**) Linear discriminative analysis (LDA) score of differentially enriched bacterial genera obtained from LEfSe analysis between the MPTP (red) and MPTP + Yam (blue) mice (with LDA score > 3 and *p*-value < 0.05, determined using Kruskal–Wallis test). (**E**) Heatmap of Spearman’s correlation analysis between the differential expressed bacterial genera and pathological markers of PD mice. Red labels indicate taxa significantly associated with PD-related indices. Data of (**A**) presented as mean ± SEM. * *p* < 0.05, ** *p* < 0.01, and *** *p* < 0.001, ns, not significant. *p*-values were calculated by one-way ANOVA with Tukey’s or Tamhane’s tests.

Emerging evidence suggests that dysbiosis of the gut microbiota is intricately associated with the occurrence and development of PD [28,37]. We next investigated the potential effects of yam supplementation on the composition of gut microbiota. To this end, we collected feces from three groups of mice and subjected them to 16S rRNA sequencing. The alpha diversity index was used to assess the diversity of the microbial community. We found that Ace, Chao, and Shannon were significantly increased in mice fed a yam diet compared with mice fed a normal diet (Figure 3A). PCA revealed a significant separation of the fecal microbial structure of the three groups of mice (Figure 3B). Similarly, principal coordinate analysis (PCoA) and nonmetric multidimensional scaling (NMDS) based on unweighted Unifrac distance also showed significant differences (Figure 3B). In addition, we also compared the relative abundance of microorganisms at different taxonomic levels between groups. Compared with the MPTP group, the top OTUs in relative abundance showed enrichment of *Bacteroidetes* and reduction in Firmicutes in MPTP + Yam mice (Figure 3C). At the genus level, the relative abundance of the intestinal microbiota in the three groups of mice also changed significantly. The most striking one was that *Ileibacterium* was almost undetectable in MPTP mice compared with the Control group, which was consistent with previous study [38]. However, supplementation with yam significantly increased the relative abundance of *Ileibacterium* in MPTP mice (Figure 3C). Further, we performed linear discriminant analysis effect size (LEfSe) analysis (log LDA score > 3) to distinguish unique bacterial phylotypes that were altered by yam administration. Compared with MPTP mice, the relative abundance of *Blautia* and *Lachnospiraceae_NK4A136_group* was significantly increased in MPTP + Yam group mice (Figure 3D).

To further understand the impact of changes in the gut microbiota on the development of PD. We associated the differentially expressed bacterial genera that were significantly altered in MPTP mice after yam intervention with PD phenotypes, including behavioral indices, striatal dopamine and its metabolite concentrations, and serum validation factor levels (Figure 3E). The correlation heat map showed that the abundance of *Bifidobacterium* and *Ileibacterium* was significantly positively correlated with the time spent on the rotating rod, the inverted grid hanging time, the levels of striatal dopamine and its metabolites, and the anti-inflammatory factor IL-10, while it was significantly negatively correlated with the climbing time, hindlimb scores, and the pro-inflammatory cytokines LPS, TNF-α, IL-1β, and IL-6. In addition, *Allobaculum*, *Lachnospiraceae_NK4A136_group*, and *Blautia* also showed negative correlations with the severity of PD pathology (Figure 3E). Collectively, these results suggest that dietary supplementation with yam may affect the balance of intestinal flora and thus ameliorate the PD pathology.

### 3.4. Yam Changed the Fecal Metabolite Profiles in MPTP-Induced PD Mice

**Figure 4:**
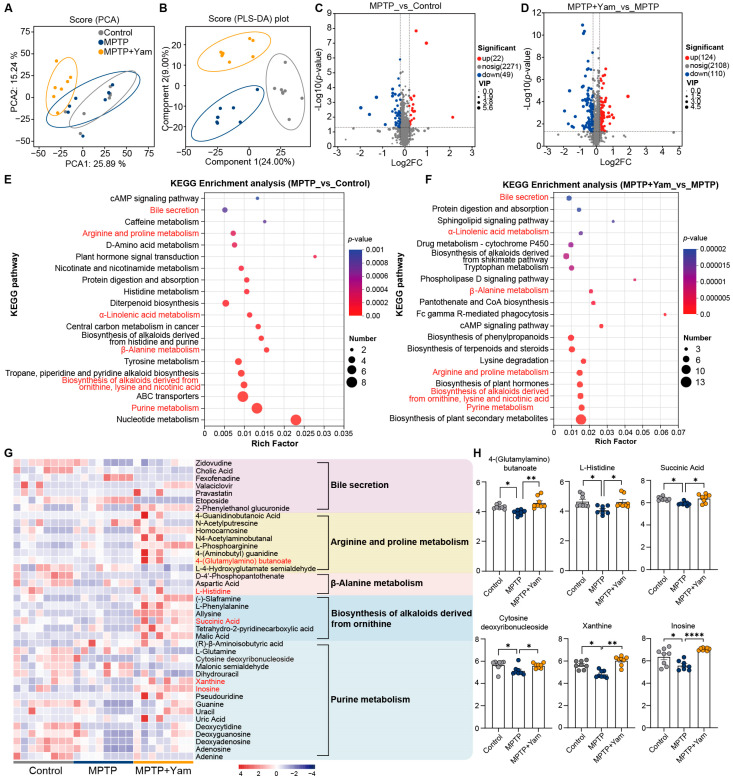
Metabolomic analysis of colonic contents in MPTP-induced PD mice treated with yam. (**A**,**B**) PCA plot and PLS-DA plot showing distinct clustering of fecal metabolites among control, MPTP, and MPTP + Yam groups (*n* = 8 mice per group) (colored ellipses represent the 80% confidence intervals for each group). (**C**,**D**) Volcano plots of the significantly differential metabolites between the MPTP group and Control group, as well as between the MPTP + Yam group and MPTP group (*p*-value < 0.05, |log2 fold change| > 1). Dashed lines indicate reference boundaries, and the points represent individual metabolites. (**E**) Bubble plot of KEGG pathway enrichment analysis for differential metabolites between MPTP and control groups. (**F**) Bubble plot of KEGG pathway enrichment analysis for differential metabolites between MPTP + Yam and MPTP groups. (**G**) Clustering heatmap of significantly differential metabolites in the KEGG pathways related to bile secretion, arginine and proline metabolism, β-alanine metabolism, alanine aspartate and glutamate metabolism, and purine metabolism among the Control, MPTP, and MPTP + Yam groups of mice. (**H**) Relative abundance of the representative differential metabolites among the Control, MPTP, and MPTP + Yam groups of mice (*n* = 8 mice per group). Data of (**H**) presented as mean ± SEM. * *p* < 0.05, ** *p* < 0.01, and **** *p* < 0.0001. *p*-values were calculated by one-way ANOVA with Tukey’s or Tamhane’s tests.

Small molecule metabolites derived from commensal gut microbiota could transmit signals beyond the gastrointestinal tract to modulate brain function and host behavior [39]. Based on the significant effects of yam treatment on gut microbiota, we hypothesized that yam may alter gut-derived signals associated with brain communication, thereby mediating pathological symptoms in MPTP-induced mice. Therefore, we conducted untargeted metabolomic analysis on colonic contents from Control, MPTP, and MPTP + Yam mice, and identified a total of 2342 metabolites in both positive and negative ion modes. PCA and PLS-DA analysis revealed a distinct separation in the clustering patterns of fecal metabolites in MPTP mice following yam intervention (Figure 4A,B). Compared to the control group, 71 metabolites including 22 upregulated and 49 downregulated metabolites exhibited significant alterations in MPTP mice (Figure 4C). Moreover, yam intervention induced the alteration of 234 fecal metabolites in MPTP-treated mice, with 124 being upregulated and 110 downregulated (Figure 4D). These differential metabolites were primarily associated with fatty acids, phospholipids, eicosanoids, carboxylic acids, amino acids, and monosaccharides. Further KEGG pathway analysis of the differentially expressed metabolites indicated that the metabolic alterations induced by MPTP were predominantly enriched in nucleotide metabolism, purine metabolism, and ABC transporters pathway (Figure 4E). Additionally, the differential metabolites of MPTP mice were observed to be enriched in bile secretion, arginine and proline metabolism, α-linolenic acid metabolism, β-alanine metabolism, and the biosynthesis of alkaloids derived from ornithine, lysine, and nicotinic acid, and purine metabolism pathway compared with the control mice (Figure 4E). Notably, yam-induced differential metabolites were also annotated in these pathways (Figure 4F). Analysis of the expression levels of differential metabolites enriched in these pathways showed that the levels of 4-(glutamylamino) butanoate, L-histidine, and succinic acid were significantly decreased in MPTP mice compared with the control group, while these levels were significantly restored after yam intervention (Figure 4G,H). 4-(glutamylamino) butanoate, an intermediate in glutamate metabolism, is involved in the synthesis and regulation of the neurotransmitter GABA [40]. Although direct evidence linking 4-(glutamylamino) butanoate to PD is lacking, GABA has been widely demonstrated to be inversely associated with PD risk, thereby partially validating the observed reduction in 4-(glutamylamino) butanoate levels in MPTP mice. Consistent with our findings, metabolomic analyses of fecal and plasma samples from PD patients have revealed significantly decreased succinic acid levels compared to healthy controls [41,42]. Importantly, yam intervention significantly attenuated MPTP-induced reductions in xanthine and inosine levels of colonic contents (Figure 4G,H). The role of purine metabolism in PD has been extensively studied, with multiple studies reporting a negative correlation between PD severity and purine metabolites, including xanthine and inosine [43,44]. Therefore, these results collectively suggest that dietary supplementation with yam effectively modulates gut microbial metabolite composition in MPTP-induced PD mice, and the enrichment of purine metabolites may contribute to the neuroprotective mechanisms of yam.

### 3.5. Yam Improves Mitochondrial Biogenesis and Inflammation in SNc of MPTP-Induced PD Mice

**Figure 5:**
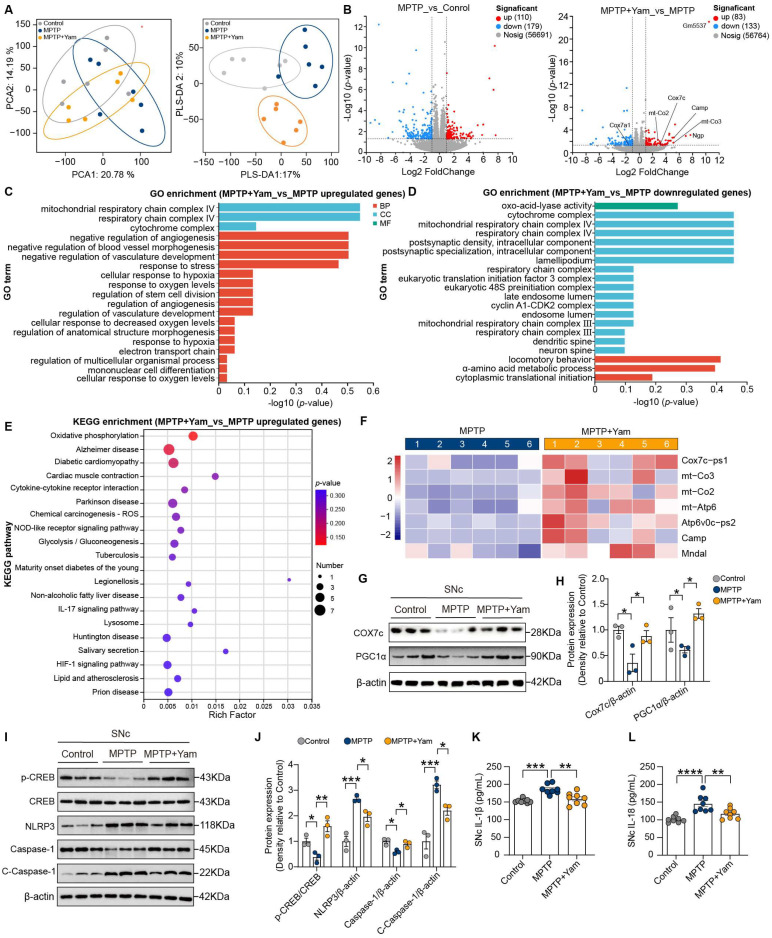
Yam-induced transcriptome profile changes improve mitochondrial dysfunction and neuroinflammation in the SNc of MPTP-induced PD mice. (**A**) PCA and PLS-DA of all transcriptome profiles in the SNc of control, MPTP, and MPTP + Yam mice (*n* = 6 per group) (colored ellipses represent the 80% confidence intervals for each group). (**B**) Volcano plots of the DEGs between the MPTP group and Control group, as well as between the MPTP + Yam group and MPTP group (*p*-value < 0.05, |log2 fold change| > 1). Dashed lines indicate reference boundaries, and the points represent individual genes. (**C**) GO enrichment analysis of upregulated DEGs between MPTP and Control groups. (**D**) GO enrichment analysis of downregulated DEGs between MPTP + Yam and MPTP groups. (**E**) Bubble plot of KEGG pathway enrichment analysis for up-regulated DEGs between MPTP + Yam and MPTP groups. (**F**) Heat map of DEGs enriched in oxidative phosphorylation and NOD-like receptor signaling pathways in MPTP and MPTP + Yam mice. The numbers represent six different samples from the MPTP and MPTP + Yam groups. (**G**,**H**) Representative Western blots and quantification analysis of COX7a1and PGC1α in the SNc of Control, MPTP, and MPTP + Yam group mice (*n* = 3 mice per group). (**I**,**J**) Representative Western blots and quantification analysis of p-CREB, CREB, NLRP3, Caspase-1, and Cleaved Caspase-1 in the SNc of Control, MPTP, and MPTP + Yam group mice (*n* = 3 mice per group). (**K**,**L**) SNc tissue IL-1β and IL-18 levels (*n* = 8 mice per group). Data of (**H**,**I**,**K**) presented as mean ± SEM. * *p* < 0.05, ** *p* < 0.01, *** *p* < 0.001, and **** *p* < 0.0001. *p*-values were calculated by one-way ANOVA with Tukey’s or Tamhane’s tests.

Based on the observed alterations in gut microbiota and systemic metabolic profiles, we further sought to elucidate how the systemic improvements induced by yam translate into local neuroprotective effects within the SNc through comprehensive RNA-seq analysis. After aligning 187.31 Gb RNA-seq reads with the *Mus musculus* genome, we detected 32,855 genes, including 32,464 known genes and 391 newly predicted genes. PCA and PLS-DA revealed significant separation in gene expression patterns among the Control, MPTP, and MPTP + Yam groups (Figure 5A). Differential expression analysis showed that, compared to the Control, 110 genes were upregulated and 179 were downregulated in the MPTP group (Figure 5B). Furthermore, we identified 216 differentially expressed genes (DEGs) in the MPTP + Yam vs. MPTP comparison, with 83 upregulated and 133 downregulated.

To characterize the biological pathways disrupted by MPTP, GO and KEGG enrichment analyses were performed on the DEGs from the MPTP vs. Control comparison. Significant alterations were observed across molecular functions, cellular components, and biological processes following MPTP treatment (Figure S1A,B). Notably, further KEGG enrichment analysis revealed that downregulated DEGs were significantly enriched in neuroactive ligand–receptor interaction and the cAMP signaling pathway, and upregulated DEGs were related to JAK-STAT signaling pathway, which mediates neuroinflammation (Figure S1C,D). For MPTP + Yam vs. MPTP comparison, GO enrichment analysis showed that upregulated DEGs in the SNc of the MPTP + Yam group were primarily enriched in mitochondrial respiratory chain complex IV-related pathways, largely attributed to the upregulation of the *Cox7c* gene (Figure 5B,C). *Cox7c* encodes a critical subunit of mitochondrial complex IV, playing a vital role in energy metabolism and oxidative phosphorylation [45]. Similarly, KEGG analysis revealed that yam promoted the oxidative phosphorylation pathway by upregulating mitochondrial genes, including *Cox7c-ps1*, *mt-Co2*, *mt-Co3*, *mt-Atp6*, and *Atp6v0c-ps2* (Figure 5E,F).

To validate the key pathways suggested by the transcriptomic analyses, Western blot analysis was performed to assess the expression of representative proteins (Figure 5G,H). Consistent with RNA sequencing data, we found that dietary yam supplementation significantly upregulated COX7c protein expression. PGC1α is a master regulator of mitochondrial biogenesis and energy metabolism that drives COX7 transcriptional activation [46]. In addition, yam intervention enhanced the activation of PGC1α. In summary, yam may promote PGC1α-mediated oxidative phosphorylation, thereby counteracting MPTP-induced mitochondrial dysfunction.

Additionally, upregulated DEGs in the MPTP + Yam group were significantly enriched in the NOD-like receptor signaling pathway, primarily due to the upregulation of cAMP-related genes. Yam supplementation promoted the activation of the CREB signaling, which subsequently inhibited NLRP3 inflammasome activation and the cleavage of Caspase-1 (Figure 5I,J). The reduced levels of IL-1β and IL-18 in SNc confirmed these anti-inflammatory effects (Figure 5K,L). In summary, these data indicates that the restoration of mitochondrial oxidative phosphorylation and the suppression of inflammatory responses in the SNc are associated with the yam-induced alleviation of motor deficits.

### 3.6. Integrated Multi-Omics Analysis on Dietary Yam Intervention

**Figure 6:**
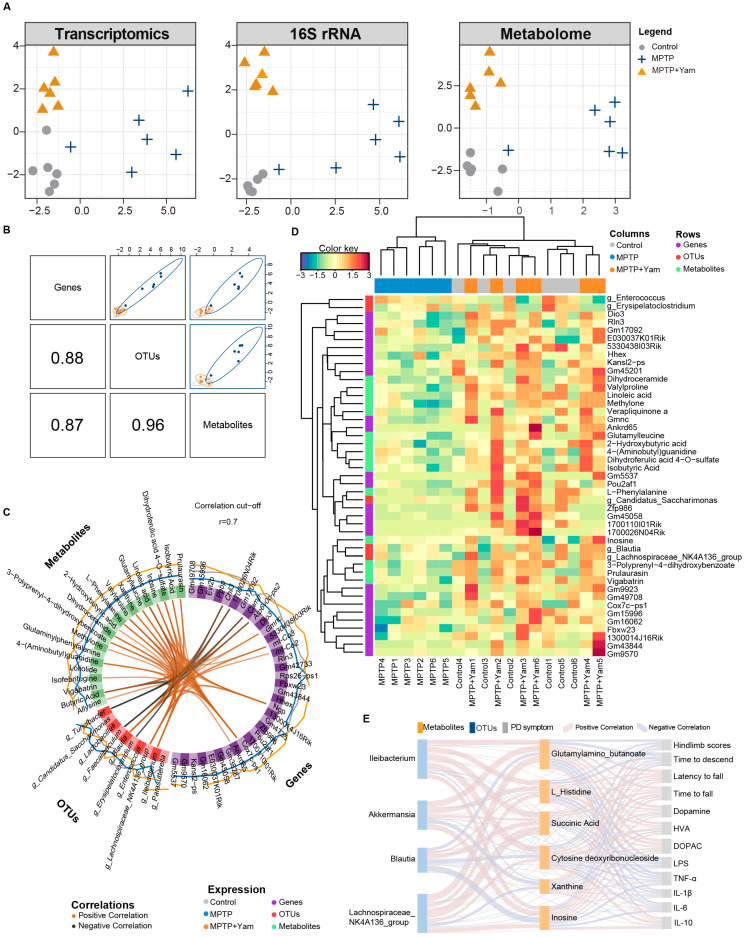
Integration of the three omics using mixOmics and DIABLO. (**A**) PLS-DA sample plots derived from the final DIABLO model for each omics dataset. (**B**) Scatterplot matrix (generated using plotDiablo) displaying the first component of each dataset (upper diagonal) and the Pearson correlation between components (lower diagonal). Samples are represented by 95% confidence ellipses. Correlation coefficients between the first components of each dataset are indicated in the lower left. (**C**) Circos plot illustrating the most significant variables and their pairwise correlations. Variables are arranged on the circumference and colored by data type (OTUs, Genes, or Metabolites). Internal connecting lines represent correlations between variables (Orange: positive; Black: negative). (**D**) CIM depicting the multi-omics molecular signatures. Columns represent samples, and rows represent the 45 most discriminatory variables selected by the model (top 15 from each dataset). Hierarchical clustering was performed on both rows and columns using Euclidean distance and complete linkage. (**E**) Sankey diagram visualizing the correlation network among key metabolites (orange), gut microbiota genera (blue), and behavioral phenotypes (gray). Link width is proportional to the correlation strength, with red indicating positive correlations and blue indicating negative correlations.

The molecular changes induced by yam intervention encompass three major levels—gut microbiota ecology, metabolite profile and host gene expression—and these three types of omics signals often exhibit potential synergistic regulation and functional coupling, relying solely on single-omics analysis is insufficient to reveal their systemic mechanisms of action. To systematically evaluate the multi-level biological effects of yam on MPTP-induced PD mice, we further integrated and analyzed 16S rRNA microbiota, non-targeted metabolomics and transcriptomic data. We employed the DIABLO framework from mixOmics to jointly model different omics data. This method enables multivariate dimensionality reduction discriminant analysis across multiple omics, thereby identifying key feature combinations that are highly correlated across omics and possess biological significance. Before the formal integrated analysis, we screened core variables closely related to yam intervention from each type of omics data, including the top 10 significantly enriched dominant bacterial genera, 124 significantly upregulated differentially expressed metabolites and 83 differentially expressed hub genes upregulated in the SNc of MPTP mice treated with yam. These variables constitute the input features of the DIABLO model, used to capture the most critical systemic signals of yam intervention. Predictive modeling employed PLS-DA combined with a repeated double cross-validation framework (rdCV-PLSDA). The results showed that all three omics groups could clearly distinguish the MPTP group and the MPTP + Yam group in the first potential component, with the metabolome exhibiting the strongest discriminative ability, suggesting that metabolite changes are a sensitive indicator in the yam response process (Figure 6A). The DIABLO model showed high consistency in the first potential component constructed from the three omics data (transcriptome, OTUs, and metabolites). The component correlation matrix showed that the correlation coefficient between transcriptome and OTUs was 0.88, between transcriptome and metabolites was 0.87, and between OTUs and metabolites reached 0.96, all showing extremely strong positive correlations (Figure 6B). These results indicate that molecular changes at different omics levels exhibit a coordinated response with consistent direction under yam intervention. Circos plot revealed significant correlations among the three most important omics features in the DIABLO model. The circular structure simultaneously presented cross-omics and intra-omics association patterns, with red lines representing strong positive correlations and blue lines representing strong negative correlations (Figure 6C). In cross-omics associations, the abundance of the *Lachnospiraceae NK4A136 group* and *Ileibacterium* showed positive correlations with metabolites such as inosine, xanthine, succinic acid, and L-histidine, further supporting a synergistic response between gut microbiota changes and metabolite remodeling. On the other hand, the purine metabolites inosine and xanthine showed the strongest positive correlations with genes related to mitochondrial oxidative phosphorylation, suggesting a close relationship between the restoration of purine metabolism and the improvement of energy metabolism gene expression. The Clustered Image Map (CIM) heatmap constructed using these key cross-omics features further showed that yam intervention exhibited a trend of clustering towards the healthy control group at multiple omics levels (Figure 6D). Furthermore, Sankey diagram (Figure 6E) was constructed to link specific metabolites, key bacterial genera, and PD clinical phenotypes, highlighting “metabolite–microbiota–phenotype” axis that supports the role of yam in improving PD symptoms by reshaping the gut environment. Specifically, *Ileibacterium* and *Lachnospiraceae_NK4A136_group* increased by yam intervention were closely associated with the production of inosine. The enrichment of inosine could further suppress neuroinflammation, as evidenced by reduced levels of pro-inflammatory cytokines like IL-6 and TNF-α, and preserves striatal dopaminergic integrity through elevated dopamine and TH expression, ultimately improved motor deficits. Overall, the multi-omics integrated analysis revealed that the process by which yam improves the pathological state of PD is not a single-pathway action but rather depends on its synergistic regulation of gut microbiota composition, metabolic pathways, and mitochondrial function-related genes, thereby forming a multidimensional interaction network across “microbiota–metabolites–host gene expression”.

## 4. Discussion

Accumulating evidence suggests that gut microbiota dysbiosis plays a key role in the pathogenesis of PD, which emphasizes the therapeutic potential of targeting the gut microbiome for intervention [9,28]. Notably, yam may exert beneficial effects on various phenotypes associated with neurological disorders. However, the neuroprotective potential of yam in PD and its underlying molecular mechanisms remain elusive. In this study, we systematically evaluated yam as an integrated dietary intervention in a PD mouse model and found that dietary yam supplementation significantly ameliorated MPTP-induced motor deficits and dopaminergic neuronal loss in the SNc. Moreover, yam supplementation strengthened intestinal barrier integrity and ameliorated gut epithelial damage induced by MPTP. Through 16S rRNA sequencing and metabolomic profiling, we revealed that yam remodeled the gut microbial composition and restored key metabolites associated with purine and glutamate metabolism. Concurrently, by integrating transcriptomic and molecular analyses, we found that dietary yam improved mitochondrial biogenesis and oxidative phosphorylation while attenuating inflammatory activation in the SNc. Integrated multi-omics analysis further validated that yam exerts its neuroprotective effects by modulating the microbiota–gut–brain axis. Collectively, our findings highlight the promising potential of yam as a compelling dietary modulator with the capacity to mitigate PD-related symptoms, wherein the restoration of gut microbiota homeostasis and microbial metabolite balance constitutes a central mechanism underlying its neuroprotective action.

Neuroprotective effects of whole yam in PD

Previous studies have mainly focused on bioactive compounds isolated from yam, such as diosgenin and dioscin, which have been reported to exert neuroprotective effects in animal models of PD. Specifically, recent studies suggested that diosgenin supplementation attenuates microglia-mediated neuroinflammation and dopaminergic neuronal loss by suppressing pro-inflammatory cytokines, iNOS expression, and ERK activation [47,48]. In addition, dioscin has been shown to ameliorate MPTP-induced PD pathology by restoring gut microbiota balance and regulating bile acid-mediated oxidative stress and neuroinflammation [49]. Although these findings provide valuable insights into the pharmacological actions of individual yam-derived components, they do not fully reflect the physiological complexity of dietary interventions. To date, the neuroprotective effects of dietary whole yam on PD have not been systematically investigated in vivo. In the present study, we found that daily supplementation with whole yam powder for six weeks effectively mitigated MPTP-induced motor deficits and dopaminergic neuronal degeneration in mice.

Restoration of intestinal barrier integrity

Disruption of the intestinal barrier has been recognized as an early pathological event in PD. Clinical case studies have confirmed that patients diagnosed with early-stage PD exhibit markedly elevated intestinal permeability and increased exposure to gut bacteria and bacterial endotoxins, indicating early impairment of barrier function [50]. Consistent with these clinical observations, histological examination in our study revealed that MPTP-induced PD mice showed severe epithelial disruption, villus atrophy, and crypt disorganization, whereas yam treatment restored mucosal continuity and villus morphology. Consistently, immunofluorescence and Western blot analyses showed that yam significantly increased the expression of tight-junction proteins, including ZO-1 and occludin, and reduced intestinal permeability, indicating improved epithelial integrity and function. Moreover, yam supplementation also reduced the MPTP-induced elevations in serum LPS and pro-inflammatory cytokines, including TNF-α and IL-6, as well as increased anti-inflammatory cytokines IL-10 level, thereby alleviating systemic inflammation associated with barrier leakage. These results suggest that yam preserves intestinal integrity by restoring epithelial morphology and enhancing the expression of tight-junction proteins, thereby maintaining barrier function and limiting luminal leakage.

Gut microbiota remodeling and functional implications

In the present study, yam supplementation further reshaped the gut microbial landscape in MPTP-induced PD mice, elevating overall microbial richness and diversity, as evidenced by increased ACE, Chao1, and Shannon indices, as well as producing distinct clustering patterns in PCA, PCoA, and NMDS analyses. Notably, one of the most striking taxonomic shifts observed in our study was the restoration of *Ileibacterium*. This genus was nearly depleted in MPTP-treated mice but was robustly reinstated to control-like levels following yam supplementation. Previous studies have repeatedly documented a marked loss of *Ileibacterium* in both MPTP- and rotenone-induced PD mouse models [38,51], suggesting that its recovery in our study may reflect a correction of PD-associated microbial dysbiosis and serve as an indicator of a stabilized gut ecological landscape. Moreover, yam intervention also enriched additional taxa, including *Blautia* and members of the *Lachnospiraceae_NK4A136_group*, as revealed by LEfSe analysis. This observation aligns with human studies reporting reduced *Blautia* abundance in PD, and with preclinical evidence that supplementation with the butyrate-producing species *Blautia* ameliorates motor deficits and restores butyrate levels in PD model mice, thereby supporting the potential functional relevance of *Blautia* restoration [52]. Likewise, increased abundance of *Lachnospiraceae* taxa has also been associated with reduced neuroinflammatory responses and improved motor performance in preclinical PD models [53,54]. To better understand the physiological significance of these microbial alterations, we integrated microbiota data with behavioral indices, nigrostriatal dopamine levels, and systemic inflammatory markers. Correlation analyses revealed that *Bifidobacterium*, *Ileibacterium*, *Lachnospiraceae_NK4A136_group*, and *Blautia* were positively associated with improved motor performance and higher striatal dopamine and metabolite concentrations, while exhibiting strong negative correlations with hindlimb rigidity scores and pro-inflammatory factors such as LPS, TNF-α, IL-1β, and IL-6. These patterns suggest functional links between yam-driven microbial shifts and amelioration of both motor dysfunction and neuroinflammation. Although the precise mechanisms by which yam modulates these beneficial taxa remain to be elucidated, the restoration of several taxa disrupted by MPTP treatment highlights that gut microbiota as a plausible mediator of yam’s neuroprotective effects. Together, our findings support that dietary yam rebalances the gut microbial ecosystem that may contribute to the prevention or attenuation of PD progression.

Microbiota-derived metabolic signaling modulating

Metabolic signaling represents a critical route through which the gut microbiota communicates with the host, particularly via microbially derived metabolites that modulate neuronal and immune pathways along the microbiota–gut–brain axis. Consistent with the alterations in microbial composition, yam supplementation also remodeled the gut-derived metabolic landscape in MPTP-induced PD mice. Our untargeted metabolomic profiling revealed widespread disruptions in key metabolic pathways following MPTP administration—including purine metabolism, amino acid turnover, and intermediates of the TCA cycle—all of which were partially normalized by yam intervention. Among the altered pathways, perturbations in purine metabolism were particularly notable. Xanthine and inosine, two metabolites repeatedly implicated in PD pathology, were significantly reduced in MPTP-treated mice but restored following yam supplementation. In a study involving 217 unmedicated PD subjects and 26 healthy controls, cerebrospinal fluid analysis demonstrated that the CSF xanthine/HVA ratio—which reflects the balance between purine turnover and dopamine metabolism—was markedly higher in PD patients compared with healthy controls [55]. Longitudinal sampling conducted approximately 24 months later in the same PD cohort revealed a further increase in this ratio. These clinical observations support the notion that perturbations in purine metabolism are characteristic biochemical features of PD. Preclinical studies have shown that inosine exerts dopaminergic neuroprotective effects in MPTP-induced Parkinsonian animal models, partly via BDNF upregulation and suppression of inflammasome activation [56]. Motivated by these findings, two large randomized clinical trials SURE-PD (phase II, 2014) and SURE-PD3 (phase III, 2021), respectively, evaluated oral inosine in early PD and demonstrated that the treatment reliably increases serum and cerebrospinal fluid urate levels [57,58], a biomarker that prospective cohort studies have identified as an inverse risk factor for PD, indicating adequate engagement of the intended biochemical target.

Mitochondrial dysfunction and its restoration by yam

Mounting evidence suggests that mitochondrial dysfunction, particularly inhibition of electron transport chain complex and the accumulation of oxidative stress, is increasingly recognized as a critical contributor to dopaminergic neurodegeneration in PD [59]. In this study, transcriptome analysis showed that yam supplementation restored the expression of several oxidative phosphorylation-related genes in the SNc of MPTP-induced PD mice, including *Cox7c*, *mt-Co2*, *mt-Co3*, and *mt-Atp6*, while concurrently downregulating the expression of *Cox7a1*. *Cox7c* is nuclear-coding subunits of cytochrome c oxidase (complex IV), involved in the assembly and stability of complex IV, and is core component in maintaining mitochondrial respiratory chain efficiency [60]. Consistent with our findings, previous study has documented a pervasive decrease in Cox7c in the brains and peripheral tissues of patients with neurodegenerative diseases, a defect that is intrinsically linked to impaired mitochondrial respiration and exacerbated oxidative stress within the central nervous system [61]. Moreover, *Cox7c* is not typically studied as an individual pathogenic driver of PD model but as part of the oxidative phosphorylation gene module regulated by PGC-1α [62]. Consistent with this, we observed that the restoration of Cox7c occurred concurrently with PGC1α activation. It is worth emphasizing that a previous systems biology study integrating gene expression data from 17 groups of PD patients, including 322 post-mortem SNc samples and 88 peripheral blood samples, showed that impaired expression of PGC-1α and its regulated mitochondrial genes is a core event in the early pathogenesis of PD [62]. Given that dysregulation of PGC-1α and its regulatory network is considered a key biochemical feature of early PD pathogenesis, the normalizing effect of yam on this pathway may contribute to the restoration of mitochondrial homeostasis. Our integrated multi-omics analysis further reveals that the restoration of mitochondrial oxidative phosphorylation function may be mainly attributed to the remodeling of purine metabolites xanthine. Although direct evidence linking xanthine to the regulation of PGC-1α remains limited, research involving other stress models—such as high glucose-induced injury in gastric epithelial cells—suggests that xanthine oxidoreductase inhibitors facilitate the intracellular accumulation of xanthine via the HPRT1-dependent purine salvage pathway. This process subsequently drives the AMPK-PGC-1α signaling axis, ultimately alleviating oxidative damage [63]. In addition, the neuroprotective effect of yam is also reflected in the inhibition of neuroinflammation. Our results showed that yam was associated with the activation of CREB expression and the inhibition of the NLRP3 inflammasome. Interestingly, inosine, as an agonist of adenosine A2A and A3 receptors, has been shown to effectively induce adenylate cyclase to produce cAMP, thereby activating PKA [64]. Crucially, activated PKA can directly phosphorylate NLRP3 protein, thereby blocking the assembly of the inflammasome and its cleavage of Caspase-1. Previous studies in PD animal models have also shown that inosine supplementation can significantly reduce the release of pro-inflammatory factors such as IL-1β [64,65].

In summary, our findings suggest that dietary yam supplementation may alleviate PD-like pathology, potentially through modulation of intestinal barrier function, gut microbiota composition, purine and energy metabolism, as well as mitochondrial and inflammatory pathways. Despite these findings, several limitations should be acknowledged. First, comparisons with other dietary interventions were not performed, and future studies are needed to determine the relative specificity and efficacy of yam supplementation. Second, the MPTP-induced model represents an acute neurotoxic paradigm and does not fully recapitulate the progressive and α-synuclein-related pathology of human PD. In addition, gut microbiota composition is influenced by multiple host and environmental factors, and thus potential inter-individual variability should be considered when interpreting the generalizability of these findings. Overall, yam may represent a potential dietary modulator of the microbiota–gut–brain axis in PD-related pathology.

## 5. Conclusions

This study indicates that dietary yam intervention effectively ameliorates motor deficits and dopaminergic neuronal loss in MPTP-induced PD mice. Yam reshapes the gut microbiota, enriching beneficial taxa like *Lachnospiraceae NK4A136* and *Ileibacterium*, and restores neuroprotective metabolites such as inosine and succinic acid. These systemic changes activate the PGC1α and COX7c expression to enhance mitochondrial biogenesis and trigger the CREB expression and significantly suppress NLRP3-mediated neuroinflammation. Overall, yam may serve as a potential dietary intervention targeting the microbiota–gut–brain axis to alleviate PD-associated neurodegeneration.

## Data Availability

The data supporting the findings of this study are available in the article. Supplementary Information (SI) is available.

## Supplementary Materials

The following supporting information can be downloaded at: https://www.mdpi.com/article/10.3390/nu18081208/s1, Supplementary Figure S1. Yam alters the transcriptome profile to alleviate neuroinflammation and signaling dysregulation in the SNc of MPTP-induced PD mice. (A,B) GO enrichment analysis of downregulated (A) and upregulated (B) DEGs between the MPTP and Control groups, categorized into biological process, cellular component, and molecular function. (C,D) Bubble plots of KEGG pathway enrichment analysis for downregulated (C) and upregulated (D) DEGs between the MPTP and Control groups. The y-axis represents KEGG pathways, and the x-axis represents the Rich Factor.
