# Supplementary material for: Dietary Yam (Dioscorea opposita Thunb.) Ameliorates Parkinson’s Disease in Mice via Gut Microbiota-Driven Mitochondrial Improvement and Neuroinflammation Inhibition
Source: Nutrients. 2026 Apr 11;18(8):1208. doi: 10.3390/nu18081208 (PMC13119290; doi:10.3390/nu18081208)

Supporting Information

Dietary yam ameliorates Parkinson's disease in mice via gut microbiota-driven mitochondrial improvement and neuroinflammation inhibition

Shuqing Zhang, Wenjia Pan, Chen Ma, Yinghua Luo, Li Dong, Junfu Ji, Lingjun Ma, Daotong Li[*]& Fang Chen[*]

College of Food Science and Nutritional Engineering, National Engineering Research Centre for Fruit and Vegetable Processing, Key Laboratory of Fruits and Vegetables Processing, Ministry of Agriculture, Engineering Research Centre for Fruits and Vegetables Processing, Ministry of Education, China Agricultural University, Beijing 100083, China

[*]Corresponding authors:

E-mail addresses: chenfangch@sina.com (Fang Chen), lidaotong@cau.edu.cn (Daotong Li).

Supplementary Fig. 1 Yam alters the transcriptome profile to alleviate neuroinflammation and signaling dysregulation in the SNc of MPTP-induced PD mice. (A,B) GO enrichment analysis of downregulated (A) and upregulated (B) DEGs between the MPTP and Control groups, categorized into biological process, cellular component, and molecular function. (C,D) Bubble plots of KEGG pathway enrichment analysis for downregulated (C) and upregulated (D) DEGs between the MPTP and Control groups. The y-axis represents KEGG pathways, and the x-axis represents the Rich Factor.

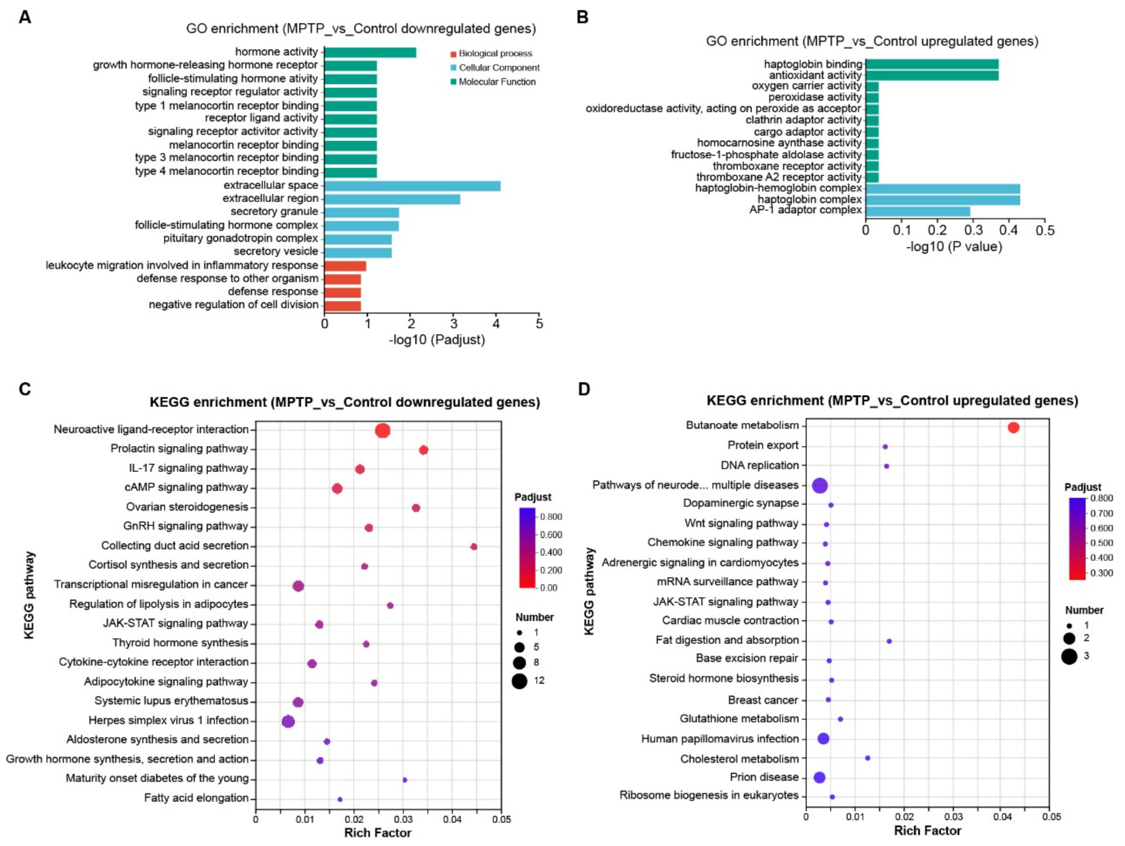

Supplement: Supplementary file 1 [file nutrients-18-01208-s001.zip › nutrients-4234995-supplementary.pdf]
